# Tetrapod distribution and temperature rise during the Permian–Triassic mass extinction

**DOI:** 10.1098/rspb.2017.2331

**Published:** 2018-01-10

**Authors:** Massimo Bernardi, Fabio Massimo Petti, Michael J. Benton

**Affiliations:** 1 MUSE—Museo delle Scienze, Corso del Lavoro e della Scienza, 3, 38122 Trento, Italy; 2 School of Earth Sciences, University of Bristol, Wills Memorial Building, Queens Road, Bristol BS8 1RJ, UK; 3 PaleoFactory – Dipartimento di Scienze della Terra, Sapienza Università di Roma, Piazzale Aldo Moro, 5, Rome 00185, Italy

**Keywords:** global warming, range expansion, palaeobiogeography

## Abstract

The Permian–Triassic mass extinction (PTME) had an enormous impact on life in three ways: by substantially reducing diversity, by reshuffling the composition of ecosystems and by expelling life from the tropics following episodes of intense global warming. But was there really an ‘equatorial tetrapod gap', and how long did it last? Here, we consider both skeletal and footprint data, and find a more complex pattern: (i) tetrapods were distributed both at high and low latitudes during this time; (ii) there was a clear geographic disjunction through the PTME, with tetrapod distribution shifting 10–15° poleward; and (iii) there was a rapid expansion phase across the whole of Pangea following the PTME. These changes are consistent with a model of generalized migration of tetrapods to higher latitudinal, cooler regions, to escape from the superhot equatorial climate in the earliest Triassic, but the effect was shorter in time scale, and not as pronounced as had been proposed. In the recovery phase following the PTME, this episode of forced range expansion also appears to have promoted the emergence and radiation of entirely new groups, such as the archosaurs, including the dinosaurs.

## 1. Introduction

The Permian–Triassic mass extinction event (PTME) was the most dramatic crisis experienced by life on Earth [1–3], and its devastating effects were felt equally on land and in the sea (e.g. [4–11]). The PTME was expressed in three ways in its effects on tetrapods: first by the sharp extinction itself, and the slow recovery thereafter; second by a deep reshuffling in the composition of ecosystems [8]; and third by the so-called ‘equatorial tetrapod gap' [12], whereby most fossil occurrences are at high latitudes, and fishes and tetrapods had apparently been driven away from the overheated tropics.

The biological impact of these poleward migrations has not been explored. These large-scale forced migrations could have played a crucial role in the recovery of life after the PTME, but such hypotheses require clarity on the timing and nature of the geographic upheavals: were the forced migrations equal to north and south? Was it one event or many? How long did the tropical expulsions last? And how did they contribute to the major biotic transitions occurring at the time?

The massive loss of biodiversity and the expulsion of taxa from the tropics are both explained by the PTME killing model, linked to the release of volcanic gases and methane stores, so producing sharp episodes of global warming, when ocean-atmospheric temperatures rose to above 40°C in the tropics, and perhaps as high, or higher, on land [12–18]. Key evidence for several episodes of sharp global warming comes from oxygen isotopes, but the absence of fishes and tetrapods from the tropical belt was also used as evidence for the warming [12].

Terrestrial tetrapods were severely hit during the PTME, with at least two-thirds of species driven to extinction [9] and the composition of tetrapod faunas changed drastically, with the disappearance of gorgonopsians and pareiasaurs, the decimation of dicynodonts and therocephalians, but also with the rise of temnospondyls, cynodonts and archosauromorphs [1,19,20]. In the earliest Triassic, a few very abundant forms, such as *Lystrosaurus* and *Procolophon*, so-called ‘disaster taxa', dominated ecosystems worldwide [7].

**Figure 1.**
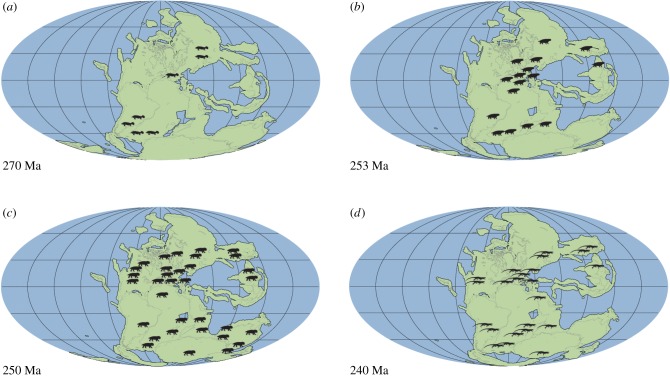
Palaeogeographic distribution of tetrapods from Middle Permian to Middle Triassic. Maps show tetrapod records, based on both skeletal and footprint data, for the (*a*) Middle Permian, (*b*) Late Permian, (*c*) Early Triassic and (*d*) Middle Triassic. Note the limited sampling in the Middle Permian, and the absence of records in the equatorial Early Triassic and the contemporaneous major expansion phase. Approximate dates in millions of years before present (Ma) shown. Silhouettes are of iconic species for that age and do not represent any specific group.

A problem with the palaeogeographic analysis is that absences could reflect gaps in the fossil record rather than real absences. In the Lower Triassic, tetrapod skeletal fossils are rare and scattered, being mainly concentrated in the South Urals of Russia and Karoo Basin of South Africa [6,7,10,21,22]. New discoveries and re-dating of fossil sites are however increasing the number of known records for this interval, and we introduce here the substantial evidence from fossilized tetrapod footprints, with their richer and geographically wider distributions, which has hitherto been ignored. In documenting the palaeogeographic distributions of tetrapods based on both skeletal and footprint data (figure 1), it can be seen how the numbers of sampled localities and regions increased through geological time, with data only from Russia and South Africa in the Middle Permian (figure 1*a*), but with increasing spread latitudinally and by regions through later time intervals (figure 1*b*–*d*).

Here, we test the hypothesis that major warming episodes associated with the PTME and with several Early Triassic isotopic spikes drove land life away from the tropics. We consider all skeletal and footprint records of tetrapods, and analyse their latitudinal distribution across the Permian–Triassic boundary (PTB) from the Middle Permian (Guadalupian) to the Middle Triassic with the aim of exploring the signature left by the PTME on the palaeobiogeography of land vertebrates. Taking into consideration the possible biases involved, we demonstrate a pronounced latitudinal shift in tetrapod distributions across the PTB, but also that there was no long-term equatorial tetrapod gap in the Early Triassic.

## 2. Methods

### (a) Database

We built a database (see electronic supplementary material) comprising tetrapod footprint occurrences at stage level from the Middle Permian (Guadalupian) to Middle Triassic. Note that by using ‘Tetrapoda' as the reference taxonomic category, we avoided all issues concerning track-trackmaker attribution, where practitioners debate which tetrapod subclade made each particular track. We also produced a database of Guadalupian to Middle Triassic tetrapod skeletal occurrences using the Paleobiology Database (PBDB, http://fossilworks.org/, downloaded 23 March 2016). We limited our research to terrestrial records (‘Environments: terrestrial' and ‘Environmental zones: lacustrine, fluvial, karst, other terrestrial' commands in PBDB's ‘included collections’ menu). We deleted from the output data all footprint occurrences. A further database using stages as time bins and Northern Hemisphere data only was also compiled to avoid the ‘Karoo basin effect' (see below).

Ages of the track- and skeleton-bearing formations were taken from the original papers and checked against the most recent available literature and the PBDB (downloaded 10 November 2016). This was done as a two-step process, first identifying the geological formations in which the fossils occurred, and then establishing age dates independently using the latest stratigraphic literature, as in the Early Tetrapod Database [23]. This avoids the problem of multiple age attributions for single geological formations, sometimes found in databases if ages are taken from fossil descriptive papers without cross-checking.

Geographic location was based on present-day GPS coordinates for the fossiliferous sites, retrieved where possible from the original papers. When not available, coordinates were derived using Google maps and Google Earth by searching the nearest locality name available approximating the locality point described in the paper. Conversion to palaeocoordinates was done by using the Paleolatitude.org online calculator [24] and its default palaeomagnetic frame [25] or retrieved from PBDB using palaeocoordinates of rock formations.

### (b) Statistical analyses

All statistical analyses were performed using R version 3.2.3 [26]. Only one entry per taxon per site was used to avoid replication of data. To evaluate statistical significance of differences between groups of latitudinal values, we used mixed effects models that account for group-specific variance, following [27]. Heterogeneity of variance was tested using the Akaike information criterion. Comparability of latitudinal distributions between different time periods was checked by dividing the fossil site or rock formation palaeolatitudes into 10-degree bins and comparing the resulting histograms with a Kolmogorov–Smirnov test. We also applied sample-based rarefaction to mediate uneven fossil sampling in different 10-degree bands. This subsampling method is widely used in both ecology and palaeobiology [28–30]. These analyses were implemented in PAST [31].

To calculate the percentage of non-marine areas for each of the epochs under study, we devised an R script (electronic supplementary material). By means of this, Mollweide palaeogeographic map files retrieved from PBDB are subdivided into 10-degree bands starting from the poles; for each band the number of non-white cells (i.e. emergent areas) is divided by the total number of cells to obtain the percentage of the global non-marine area represented by each band.

### (c) Rock formations and the ‘occupancy ratio’

By using PBDB, we produced two databases of terrestrial sedimentary formations from Guadalupian to Middle Triassic. In the first (TBF, tetrapod-bearing formations), we limited our search to tetrapod terrestrial records using the commands described above. In the second (AF, all formations), we produced a list of ‘all (known) terrestrial sedimentary formations’ without limiting the search to any specific taxon. We acknowledge that the PBDB does not include all named formations because some might have not yielded any fossils, but we postulate that most Permian–Triassic sedimentary formations in fact did (plant, invertebrate, fish, tetrapod or trace fossil), making it therefore reasonable to equate ‘all fossil-bearing formations’ with ‘all sedimentary formations’. Assuming that rock formations have limited areal extent, we also associated a (single) palaeocoordinate with each formation. Formations were then grouped into 10-degree bands; where a formation crossed two bands we assigned it to the (one) band where more records (entries) were present in PBDB. AF and TBF were then plotted against latitude to provide a visual representation of the palaeogeographical distribution of rock formations for each time bin (epoch). Finally, the TBF/AF ratio was calculated to obtain the ‘occupancy ratio'.

## 3. Results

### (a) Footprint data

**Figure 2.**
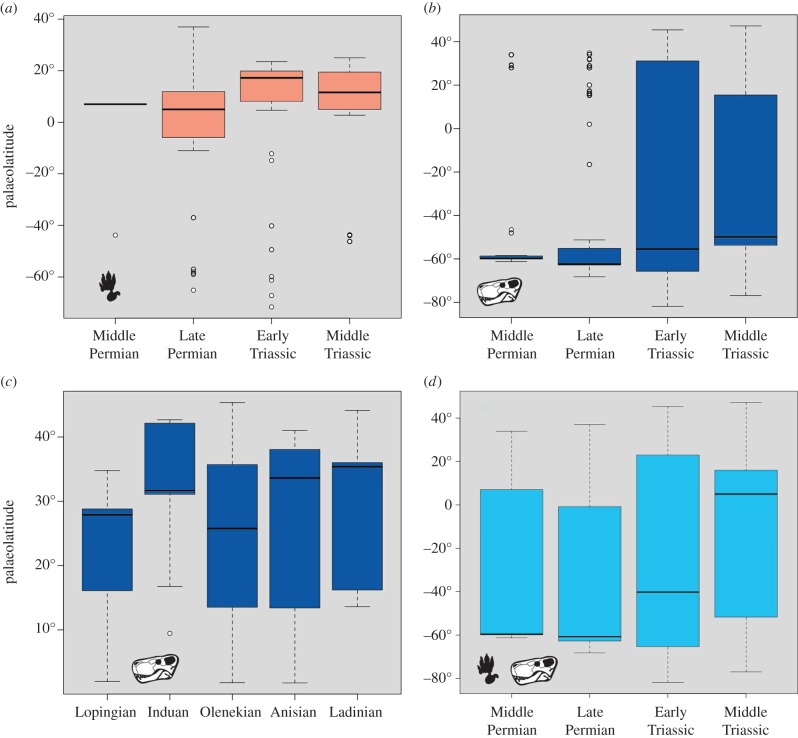
Palaeolatitudinal distributions of tetrapods from Middle Permian to Middle Triassic. (*a*) Footprint records. (*b*) Skeletal records (epoch-level time bins). (*c*) Skeletal records (stage-level time bins). (*d*) Total samples, combining footprints and skeletons.

The latitudinal distribution of ichnological data (figure 2*a*) shows that the vast majority of records are from subtropical palaeolatitudes (Middle Permian 86%, Late Permian 69%, Early Triassic 92%, Middle Triassic 94%), ranging between 15° S and 20° N. The Middle Permian ichnological record is sparse (*n* = 8), while sample sizes increase substantially in the Late Permian (*n* = 59), Early Triassic (*n* = 112) and Middle Triassic (*n* = 238). No significant difference was found between Middle and Late Permian latitudinal distributions (*t* = −0.22, *p* = 0.82), nor between the Early and Middle Triassic (*t* = −0.30, *p* = 0.76), but a significant difference (*t* = 2.97, *p* = 0.003) was found across the PTB, with the mean latitude shifting from 1° S in the Late Permian to 10° N in the Early Triassic (median from 5° N to 17° N). Mean Middle Triassic palaeolatitudes shifted back towards the equator (mean 9° N, median 11° N). We could not make these comparisons using shorter bin durations (stages), given the paucity of Induan and Ladinian records. Notably the three records of Induan footprints are from high palaeolatitudes (namely, South Africa, Australia and Antarctica), making it the only time bin considered where the average palaeolatitude of footprint data is higher than the skeletal record. However, the paucity of data prevents any statistically sound conclusion.

### (b) Skeletal data

The latitudinal distribution of skeletal data (figure 2*b*) gives an entirely different picture, showing that most records are from mid to high palaeolatitudes of the Southern Hemisphere (all means between 22° S and 47° S, medians between 49° S and 62° S). Statistical comparison of Middle (*n* = 79) and Late Permian (*n* = 291) and of Early (*n* = 405) and Middle Triassic (*n* = 400) did not find any significant difference in latitudinal distribution (*t* = −1.56, *p* = 0.11 and *t* = 1.63, *p* = 0.10, respectively). A significant shift was however found across the PTB (*t* = 6.73, *p* < 0.0001). Late Permian and Early Triassic samples have slightly different mean latitudes (from 62° S to 55° S, median from 47° S to 28° S), and very different dispersions of values (*σ* = 30.64 for the Late Permian to *σ* = 46.99 in the Early Triassic), with nearly all Late Permian data ranging from 50° S to 70° S (*Q*_1_ = 62° S, *Q*_3_ = 55° S) and Early Triassic data spread from 80° S to 50° N (*Q*_1_ = 65° S, *Q*_3_ = 31° N).

**Figure 3.**
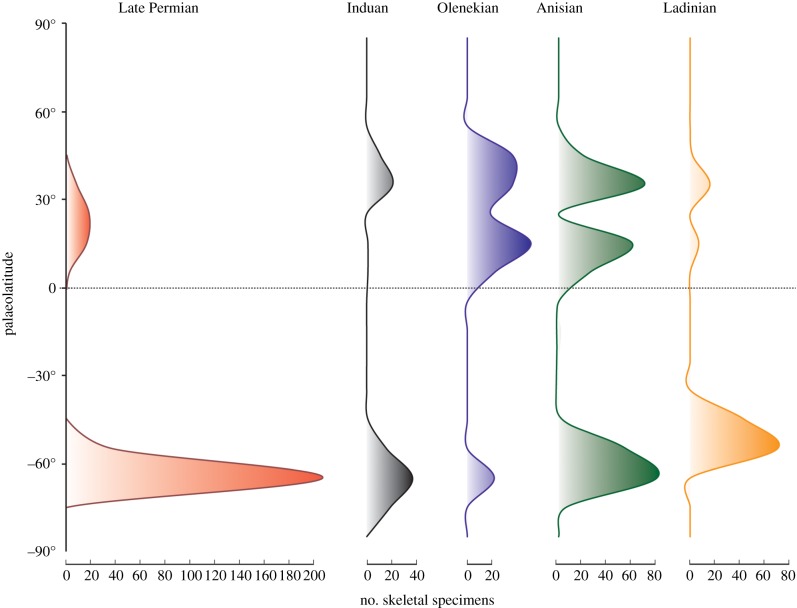
Palaeolatitudinal distribution of rarefied skeletal records. The poleward shift is constrained to the Induan.

To explore finer details in the data, the skeletal records were plotted against stage bins for the Early and Middle Triassic (figures 2*c* and 3). We considered Northern Hemisphere data only, to avoid the ‘Karoo basin effect' (i.e. the unbalanced distribution of data in the Southern Hemisphere which are nearly all from the 60° latitudinal belt). Significant differences were found (figure 2*c*) between the Lopingian and Induan (*t* = 5.99, 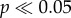) and the Induan and Olenekian (*t* = −5.63, 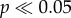), while no difference was found between the Olenekian and Anisian (*t* = −0.06, *p* = 0.94). The Lopingian and Induan distributions have widely different means (23° N and 33° N, respectively), and only slightly overlapping ranges (Lopingian: minimum value 1° N, *Q*_1_ = 16° N, *Q*_3_ = 28° N, maximum value 34° N; Induan: minimum value 9° N, *Q*_1_ = 31° N, *Q*_3_ = 42° N, maximum value 42° N). Latitudinal values for the Olenekian sample were not significantly different from those of the Lopingian (*t* = 1.22, *p* = 0.26) and they show a very similar distribution (min value 1° N, *Q*_1_ = 13° N, *Q*_3_ = 35° N, max value 45° N).

### (c) Data comparison and integration

The latitudinal distribution of footprint versus skeletal records is statistically significantly different in all time bins (electronic supplementary material). Middle and Late Permian total samples (i.e. combined footprint + skeletal; figure 2*d*) are not significantly different (*t* = −0.72, *p* = 0.46), while Late Permian and Early Triassic total samples are (*t* = 7.45, 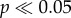). The distribution of values is similar for the Middle Permian (mean = 37° S, median 59° S, *Q*_1_ = 59° S, *Q*_3_ = 7° N) and Late Permian (mean = 40° S, median 60° S, *Q*_1_ = 62° S, *Q*_3_ = 3° S), while Early Triassic data are shifted to the north and have the highest variance (mean = 19° S, median 40° S, *Q*_1_ = 65° S, *Q*_3_ = 23° N).

**Figure 4.**
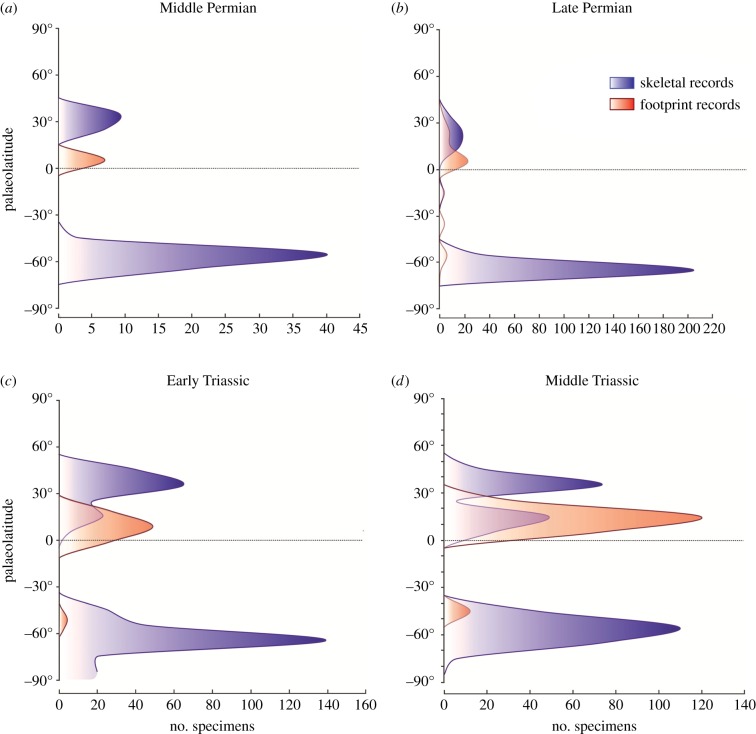
Palaeolatitudinal distribution of rarefied footprint and skeletal records. Footprint records (red curve) and skeletal records (blue curve). (*a*) Middle Permian, (*b*) Late Permian, (*c*) Early Triassic and (*d*) Middle Triassic.

The latitudinal shift across the PTB is highlighted when, after rarefaction to mediate unbalanced sampling, skeletal data only (figure 3) and all data (figure 4) are plotted.

### (d) Occupancy ratio

**Figure 5.**
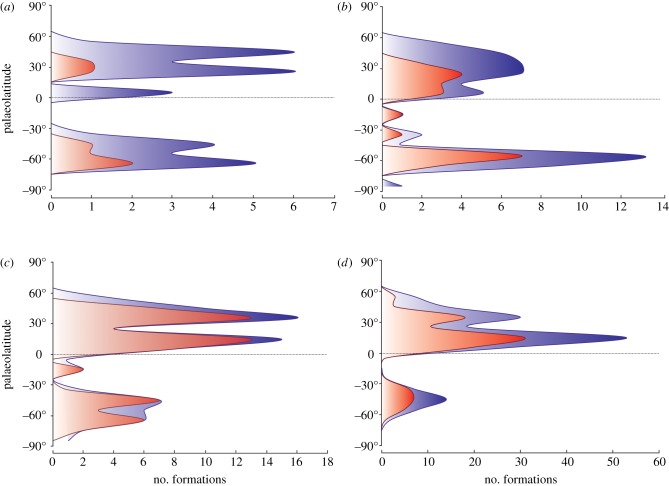
Palaeolatitudinal distribution of available rock. All formations (AF, blue curve) and tetrapod-bearing formations (TBF, red curve). (*a*) Middle Permian, (*b*) Late Permian, (*c*) Early Triassic and (*d*) Middle Triassic.

**Figure 6.**
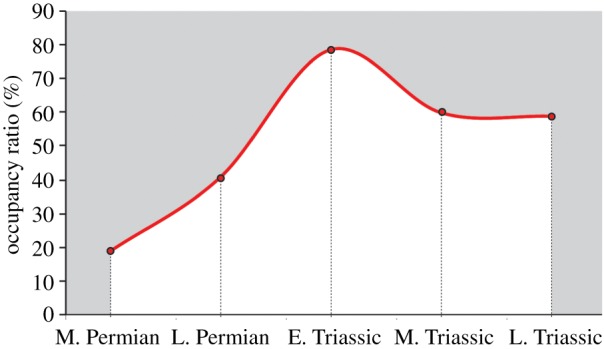
The ‘Occupancy ratio' through time. Note the peak in the Early Triassic possibly corresponding to a major tetrapod expansion phase.

Geographic distributional data for terrestrial organisms obviously depend on the availability of land, so the latitudinal distribution of all geological formations (AF) was compared with those containing tetrapod remains (TBF; figure 5). For each time bin, the curves are similar, showing that tetrapods are present, as footprints or skeletons, in a more or less constant fraction of all formations. The percentage occupancy ratio (TBF/AF %), however, varies consistently through time (figure 6), being lowest in the Middle Permian (18.75%), increasing in the Late Permian (40.67%), highest in the Early Triassic (78.57%) and decreases in the Middle Triassic (60%). A verification analysis of the Late Triassic (not discussed but provided as electronic supplementary material) resulted in a comparable 58.88%. In each time bin (figure 5), it is not clear that tetrapods are represented more heavily in any latitudinal belt, but are uniformly abundant or rare throughout.

## 4. Discussion

The 10–15° northward shift of tetrapods across the PTB was unexpected, and yet it occurs in both the ichnological and skeletal data (figure 1*a*–*d*). As analysis of the Northern Hemisphere data only demonstrates, this shift can be interpreted as a poleward shift, since Southern Hemisphere data are heavily biased by the concentration of records in the Karoo basin. The northwards shift of mean and median palaeolatitudinal data was coupled with a maximum poleward spread of tetrapods in the Early Triassic, perhaps an effect noted before [12]. This expansion phase is matched also by the high value of the occupancy ratio (figure 6) in the Early Triassic (79%), indicating maximum sampling from available rock units. The northward shift across the PTB was especially clear in the finer-scale analysis (figures 2*c* and 3), where all Induan records (mean, median, quartiles) were shifted 10–15° northward with respect to the Lopingian values. Following this northwards move, there was a reversal of the same amount in the Olenekian, and this was maintained subsequently through the Anisian and Ladinian, suggesting that the northwards move in the earliest Triassic might have been a temporary event.

This short-term northwards shift in the Induan, a time span of 0.7 Myr (251.9–251.2 Ma [32]), may reflect the ‘tetrapod equatorial gap' discovered by Sun *et al*. [12] in the Early Triassic. However, our integrated analysis, based on both skeletal and footprint records, does not confirm the suggestion that tetrapods vacated all equatorial regions throughout the Early Triassic, but that tetrapods were distributed at all latitudes across the PTB (figures 1 and 2), supporting similar recent conclusions by Romano *et al*. [33] for bony fishes.

At low latitudes, the presence of tetrapods is shown mostly by ichnological data, while skeletal records best document mid- to high-palaeolatitude localities, in both the Late Permian and the Early Triassic. Although specific studies are needed to explain this pattern, given that footprints (but not skeletal remains) are present at both high and low palaeolatitudes for each time bin, this decoupled pattern suggests that some taphonomic process biased the skeletal record. The harsh terrestrial environment of the low-latitude earliest Triassic [9,10], for example, could have been unconducive for the preservation of skeletal remains, which could have also been more frequently processed by predators.

The rapid, poleward spreading phase here documented, is consistent with a model of generalized shift of terrestrial vertebrates to higher latitudinal, possibly cooler regions [12], in a dramatic attempt to escape from the low, superhot latitudes. Even at mid- to high latitudes, tetrapods would have experienced severe climate-induced challenges, such as increasing temperatures, acidification, changes in hydrological cycles, reduced productivity and widespread wildfires [9,10,18,34], but the steep latitudinal temperature gradient which rapidly developed during the Early Triassic [35,36] might have provided access to viable refugia in the mid- to high latitudes in a context of generalized open ecospace after the extinction [1–3].

A scenario in which the PTME triggers a major spreading event might also help explain some puzzling features of late Early Triassic and early Middle Triassic tetrapod palaeogeography, such as the widespread geographic ranges of some groups soon after their origin (e.g. crown archosaurs [37–40]). If climatic belts had not yet stabilized, these clades that originated or diversified in the Early Triassic would have had to shift rapidly with changing climates, until stability returned in the Middle Triassic.

This northwards shift cannot be explained by the 0.3–0.4° per million year northward drift of Pangaea, which occurred during the Permo–Triassic transition [25,41] (see electronic supplementary material) nor by any changes in relative areas of exposed land, which did not change significantly across the PTB (electronic supplementary material). Our calculations indicate almost the same extent of land surface (about 33%) in the 0–10° N band for both Late Permian and Early Triassic, a modest 5% increase in the Early Triassic in the 10–20° N band, and more land surface in the Late Permian than in the Early Triassic in the 20–40° N band.

A key problem with this analysis, and for any such analysis of palaeogeographic distribution, is that land areas were not uniformly distributed at all palaeolatitudes. So, for example, the peaks in occurrence of tetrapods closely match peaks in the occurrence of rock units (figure 5). However, here we are comparing distributions through a relatively short span of geological time, some 40 Myr, and continents had not moved much during that time (little more than 1–2° between time bins used in our analyses). Further, while terrestrial tetrapods can generally be found only on such land areas (rare bones are washed into marine sediments), and this is entirely true for the footprints, the relative distributions of such finds do not precisely map onto the rock curves. In fact, the northernmost occurrences in each time bin lie north of the major land masses, so we are sampling to the limits of possible distributions. Southern Hemisphere sites, however, may be less well sampled, with nearly all records throughout the interval under study coming from the Karoo Basin of South Africa, and limited sampling from southern equatorial and polar regions.

One of our most surprising results was the major difference between ichnological and skeletal data (cf. figure 2*a*,*b*). The differences, however, are in means rather than overall distributions, and this reflects the rarity of footprints in the Karoo, where skeletal fossils of tetrapods are most abundant, and the relative abundance of footprints in Europe, where skeletal fossils are nearly absent. This decoupled pattern suggest that the two records are controlled by different factors, emphasizing the need to consider both, complementary sources of data when the occurrences are so patchy. It also highlights the utility of footprint data, even at very high taxonomic level, especially when the skeletal record is scarce or absent.

A key benefit of the integration was also in mitigating the massive dominance of earlier analyses by two key regions, Russia and South Africa. We have shown that tetrapods were distributed both at high and low latitudes across the PTB (contra [12]; see also [33]), that there was a clear geographic disjunction across the PTB, with tetrapod distribution shifting 10–15° northward, and during the PTME, a rapid spreading phase across the whole of Pangea is supported by both footprint and skeletal data, considered with respect to available rock formations.

These changes are consistent with a model of generalized migration of terrestrial vertebrates to higher-latitudinal, cooler regions, in an attempt to escape from the superhot climate that developed in the equatorial belt in the earliest Triassic [12]. In the aftermath of the extinction event, this episode of forced biogeographic shift might also have promoted the emergence and the radiation of entirely new groups, such as the archosaurs, including the dinosaurs.

## Supplementary Material

Dataset

## Supplementary Material

Statistics and additional data

## Data accessibility

The dataset used in statistical analyses and all statistical analyses are available as electronic supplementary material.
