# Supplementary material for: Tetrapod distribution and temperature rise during the Permian–Triassic mass extinction
Source: Proc Biol Sci. 2018 Jan 10;285(1870):20172331. doi: 10.1098/rspb.2017.2331 (PMC5784198; doi:10.1098/rspb.2017.2331)
Supplement: Statistics and additional data [file rspb20172331supp2.docx]

**Supplementary materials**

**S1 Materials and methods**

S1.1 R code for Non Marine Area calculation

getStats<-function(map,name) {

# Get number of rows in a 10° of latitude

rows<-height(map)

# 10°:180°=x:rows

separators<-19

rows.in.band<-rows/19

# Create storage for stats

stats<-rep(NA,separators)

# For every bands...

for(i in 1:(separators-1)) {

if(i==1) {

chunk<-map[,1:rows.in.band,]

} else {

# ...extracts the band from the image...

chunk<-map[,(i*rows.in.band):((i+1)*rows.in.band),]

}

# Transforms the chunk in a matrix of RGBA

chunk<-matrix(chunk,ncol=4)

# Removes the transparent cells

chunk<-chunk[-(which(chunk[,4]==0)),]

# ...counts the number of remaining cells

num<-dim(chunk)[1]

# counts the number of black cells

match<-apply(chunk, 1, identical, c(0,0,0,1))

num.black<-length(which(match))

# ...and stores the percentage of non-white pixels in a vector print(num.black/num)

stats[i]<-num.black/num

}

return(stats)

**S2 Results**

S2.1 Statistical analyses

**Kolmogorov-Smirnov**

# middle permian - late permian 10-degree lat bin kolmogorov-smirnov test (total data)

> x <- c(0,0,0,0,0,9,7,0,7,0,0,0,0,4,40,20,0,0)

> y <- c(0,0,0,0,0,11,26,25,25,1,7,0,4,0,43,208,0,0)

> ks.test (x,y)

Two-sample Kolmogorov-Smirnov test

data: x and y

D = 0.2222, p-value = 0.7658 # difference not significant

alternative hypothesis: two-sided

# late permian - early triassic 10-degree lat bin kolmogorov-smirnov test (total data)

> z <- c(0,0,0,0,41,65,45,72,32,0,2,0,1,29,46,142,22,20)

> ks.test (y,z)

Two-sample Kolmogorov-Smirnov test

data: y and z

D = 0.3333, p-value = 0.27 # difference not significant

alternative hypothesis: two-sided

# early triassic - middle triassic 10-degree lat bin kolmogorov-smirnov test (total data)

> g <- c(0,0,0,0,18,74,12,141,158,0,2,0,0,56,110,74,6,0)

> ks.test (z,g)

Two-sample Kolmogorov-Smirnov test

data: z and g

D = 0.2222, p-value = 0.7658 # difference not significant

alternative hypothesis: two-sided

**Latidutinal distribution_R comparison_Ichno VS Skeletal**

# comparison between guadalupian footprint and skeletal data

> a<-read.table("ISPmed.txt", header=TRUE, sep="\t")

> group <- as.factor(a[,2])

> M.lm <- gls(lat~group,data=a)

> vf2 <- varIdent(form=~1|group)

> M.gls <- gls(lat~group,data=a,weights=vf2)

> anova(M.lm,M.gls)

Model df AIC BIC logLik Test L.Ratio p-value

M.lm 1 3 860.9809 868.3088 -427.4904

M.gls 2 4 858.4528 868.2234 -425.2264 1 vs 2 4.528083 0.0333

> summary(M.gls)

Generalized least squares fit by REML

Model: lat ~ group

Data: a

AIC BIC logLik

858.4528 868.2234 -425.2264

Variance function:

Structure: Different standard deviations per stratum

Formula: ~1 | group

Parameter estimates:

1 2

1.000000 2.047525

Coefficients:

Value Std.Error t-value p-value

(Intercept) 42.18266 13.356968 3.158101 0.0022

group -41.51266 7.578991 -5.477333 0.0000 # significant

Correlation:

(Intr)

group -0.966

Standardized residuals:

Min Q1 Med Q3 Max

-2.4748739 -0.5179485 -0.5103345 0.3535534 2.0338137

Residual standard error: 17.96051

Degrees of freedom: 87 total; 85 residual

# comparison between lopingian footprint and skeletal data

> a<-read.table("ISPsup.txt", header=TRUE, sep="\t")

> group <- as.factor(a[,2])

> M.lm <- gls(lat~group,data=a)

> vf2 <- varIdent(form=~1|group)

> M.gls <- gls(lat~group,data=a,weights=vf2)

> anova(M.lm,M.gls)

Model df AIC BIC logLik Test L.Ratio p-value

M.lm 1 3 3369.473 3381.029 -1681.736

M.gls 2 4 3369.338 3384.747 -1680.669 1 vs 2 2.134513 0.144

> summary(M.gls)

Generalized least squares fit by REML

Model: lat ~ group

Data: a

AIC BIC logLik

3369.338 3384.747 -1680.669

Variance function:

Structure: Different standard deviations per stratum

Formula: ~1 | group

Parameter estimates:

1 2

1.000000 1.166291

Coefficients:

Value Std.Error t-value p-value

(Intercept) 44.99090 7.071589 6.362205 0

group -46.45361 3.862763 -12.026007 0 # significant

Correlation:

(Intr)

group -0.974

Standardized residuals:

Min Q1 Med Q3 Max

-2.4225716 -0.4858120 -0.4260797 0.2460253 2.6995831

Residual standard error: 26.26849

Degrees of freedom: 350 total; 348 residual

# comparison between early triassic footprint and skeletal data

> a<-read.table("ISTinf.txt", header=TRUE, sep="\t")

> group <- as.factor(a[,2])

> M.lm <- gls(lat~group,data=a)

> vf2 <- varIdent(form=~1|group)

> M.gls <- gls(lat~group,data=a,weights=vf2)

> anova(M.lm,M.gls)

Model df AIC BIC logLik Test L.Ratio p-value

M.lm 1 3 5342.870 5355.603 -2668.435

M.gls 2 4 5252.591 5269.567 -2622.295 1 vs 2 92.27951 <.0001

> summary(M.gls)

Generalized least squares fit by REML

Model: lat ~ group

Data: a

AIC BIC logLik

5252.591 5269.567 -2622.295

Variance function:

Structure: Different standard deviations per stratum

Formula: ~1 | group

Parameter estimates:

1 2

1.000000 2.385563

Coefficients:

Value Std.Error t-value p-value

(Intercept) 48.31412 4.393967 10.99556 0

group -38.19189 2.985848 -12.79097 0 # significant

Correlation:

(Intr)

group -0.944

Standardized residuals:

Min Q1 Med Q3 Max

-4.1495653 -0.7927728 -0.3432907 0.9766371 1.5629642

Residual standard error: 19.69658

Degrees of freedom: 517 total; 515 residual

# comparison between middle triassic footprint and skeletal data

> a<-read.table("ISTmed.txt", header=TRUE, sep="\t")

> group <- as.factor(a[,2])

> vf2 <- varIdent(form=~1|group)

> M.lm <- gls(lat~group,data=a)

> M.gls <- gls(lat~group,data=a,weights=vf2)

> anova(M.lm,M.gls)

Model df AIC BIC logLik Test L.Ratio p-value

M.lm 1 3 6329.656 6343.022 -3161.828

M.gls 2 4 6062.031 6079.852 -3027.016 1 vs 2 269.6246 <.0001

> summary(M.gls)

Generalized least squares fit by REML

Model: lat ~ group

Data: a

AIC BIC logLik

6062.031 6079.852 -3027.016

Variance function:

Structure: Different standard deviations per stratum

Formula: ~1 | group

Parameter estimates:

1 2

1.000000 3.036282

Coefficients:

Value Std.Error t-value p-value

(Intercept) 41.92185 2.782860 15.06430 0

group -32.42349 2.301076 -14.09058 0 # significant

Correlation:

(Intr)

group -0.954

Standardized residuals:

Min Q1 Med Q3 Max

-4.0013981 -0.6810379 0.0467469 0.8071640 1.6568283

Residual standard error: 13.93972

Degrees of freedom: 638 total; 636 residual

**Latitudinal distribution_R comparison_ICHNO**

# comparison between guadalupian and lopingian footprint data

> a<-read.table("Ipmed-psup.txt", header=TRUE, sep="\t")

> group <- as.factor(a[,2])

> vf2 <- varIdent(form=~1|group)

> M.lm <- gls(lat~group,data=a)

> M.gls <- gls(lat~group,data=a,weights=vf2)

> anova(M.lm,M.gls)

Model df AIC BIC logLik Test L.Ratio p-value

M.lm 1 3 617.6683 624.1915 -305.8342

M.gls 2 4 618.1844 626.8819 -305.0922 1 vs 2 1.483958 0.2232

> summary(M.lm)

Generalized least squares fit by REML

Model: lat ~ group

Data: a

AIC BIC logLik

617.6683 624.1915 -305.8342

Coefficients:

Value Std.Error t-value p-value

(Intercept) 2.802712 18.337271 0.1528424 0.879

group -2.132712 9.608978 -0.2219499 0.825 # not significant

Correlation:

(Intr)

group -0.985

Standardized residuals:

Min Q1 Med Q3 Max

-2.4951742 0.0475496 0.2533985 0.5004171 1.5080964

Residual standard error: 25.50415

Degrees of freedom: 67 total; 65 residual

# comparison between lopingian and early triassic footprint data

> a<-read.table("Ipsup-tinf.txt", header=TRUE, sep="\t")

> group <- as.factor(a[,2])

> M.lm <- gls(lat~group,data=a)

> vf2 <- varIdent(form=~1|group)

> M.gls <- gls(lat~group,data=a,weights=vf2)

> anova(M.lm,M.gls)

Model df AIC BIC logLik Test L.Ratio p-value

M.lm 1 3 1541.812 1551.202 -767.9060

M.gls 2 4 1537.187 1549.707 -764.5935 1 vs 2 6.625042 0.0101

> summary(M.gls)

Generalized least squares fit by REML

Model: lat ~ group

Data: a

AIC BIC logLik

1537.187 1549.707 -764.5935

Variance function:

Structure: Different standard deviations per stratum

Formula: ~1 | group

Parameter estimates:

1 2

1.0000000 0.7498189

Coefficients:

Value Std.Error t-value p-value

(Intercept) -13.04766 7.088421 -1.840700 0.0674

group 11.58494 3.893500 2.975457 0.0034 # significant

Correlation:

(Intr)

group -0.973

Standardized residuals:

Min Q1 Med Q3 Max

-4.1495630 -0.1001306 0.2923231 0.4943886 1.4642166

Residual standard error: 26.26846

Degrees of freedom: 171 total; 169 residual

# comparison between early triassic and middle triassic footprint data

> a<-read.table("Itinf-tmed.txt", header=TRUE, sep="\t")

> group <- as.factor(a[,2])

> vf2 <- varIdent(form=~1|group)

> M.lm <- gls(lat~group,data=a)

> M.gls <- gls(lat~group,data=a,weights=vf2)

> anova(M.lm,M.gls)

Model df AIC BIC logLik Test L.Ratio p-value

M.lm 1 3 2933.603 2945.160 -1463.802

M.gls 2 4 2916.299 2931.708 -1454.149 1 vs 2 19.30384 <.0001

> summary(M.gls)

Generalized least squares fit by REML

Model: lat ~ group

Data: a

AIC BIC logLik

2916.299 2931.708 -1454.15

Variance function:

Structure: Different standard deviations per stratum

Formula: ~1 | group

Parameter estimates:

1 2

1.0000000 0.7077237

Coefficients:

Value Std.Error t-value p-value

(Intercept) 10.746103 3.830404 2.8054750 0.0053

group -0.623871 2.068898 -0.3015474 0.7632 # not significant

Correlation:

(Intr)

group -0.977

Standardized residuals:

Min Q1 Med Q3 Max

-4.1495652 -0.2509633 0.1536355 0.5131738 1.1120469

Residual standard error: 19.69658

Degrees of freedom: 350 total; 348 residual

**Latitudinal distribution_R comparison_SKELETAL**

# comparison between guadalupian and lopingian skeletal data

> a<-read.table("Spmed-psup.txt", header=TRUE, sep="\t")

> group <- as.factor(a[,2])

> M.lm <- gls(lat~group,data=a)

> vf2 <- varIdent(form=~1|group)

> M.gls <- gls(lat~group,data=a,weights=vf2)

> anova(M.lm,M.gls)

Model df AIC BIC logLik Test L.Ratio p-value

M.lm 1 3 3611.991 3623.716 -1802.996

M.gls 2 4 3609.607 3625.239 -1800.803 1 vs 2 4.384705 0.0363

> summary(M.gls)

Generalized least squares fit by REML

Model: lat ~ group

Data: a

AIC BIC logLik

3609.607 3625.239 -1800.803

Variance function:

Structure: Different standard deviations per stratum

Formula: ~1 | group

Parameter estimates:

1 2

1.0000000 0.8330944

Coefficients:

Value Std.Error t-value p-value

(Intercept) -33.76899 8.467579 -3.988034 0.0001

group -7.07366 4.510440 -1.568287 0.1177 # not significant

Correlation:

(Intr)

group -0.981

Standardized residuals:

Min Q1 Med Q3 Max

-0.6617448 -0.4920137 -0.4783047 -0.2379067 2.6995831

Residual standard error: 36.77459

Degrees of freedom: 370 total; 368 residual

# comparison between lopingian and early triassic skeletal data

> a<-read.table("Spsup-tinf.txt", header=TRUE, sep="\t")

> group <- as.factor(a[,2])

> M.lm <- gls(lat~group,data=a)

> vf2 <- varIdent(form=~1|group)

> M.gls <- gls(lat~group,data=a,weights=vf2)

> anova(M.lm,M.gls)

Model df AIC BIC logLik Test L.Ratio p-value

M.lm 1 3 7140.137 7153.765 -3567.069

M.gls 2 4 7084.742 7102.912 -3538.371 1 vs 2 57.39515 <.0001

> summary(M.gls)

Generalized least squares fit by REML

Model: lat ~ group

Data: a

AIC BIC logLik

7084.742 7102.912 -3538.371

Variance function:

Structure: Different standard deviations per stratum

Formula: ~1 | group

Parameter estimates:

1 2

1.000000 1.533697

Coefficients:

Value Std.Error t-value p-value

(Intercept) -67.76299 4.284066 -15.817449 0

group 19.84667 2.945650 6.737618 0 # significant

Correlation:

(Intr)

group -0.943

Standardized residuals:

Min Q1 Med Q3 Max

-1.1439305 -0.7729801 -0.4789575 0.9766369 2.6995836

Residual standard error: 30.6367

Degrees of freedom: 696 total; 694 residual

# comparison between early triassic and middle triassic skeletal data

> a<-read.table("Stinf-tmed.txt", header=TRUE, sep="\t")

> group <- as.factor(a[,2])

> M.lm <- gls(lat~group,data=a)

> vf2 <- varIdent(form=~1|group)

> M.gls <- gls(lat~group,data=a,weights=vf2)

> anova(M.lm,M.gls)

Model df AIC BIC logLik Test L.Ratio p-value

M.lm 1 3 8400.698 8414.763 -4197.349

M.gls 2 4 8398.323 8417.077 -4195.162 1 vs 2 4.37484 0.0365

> summary(M.gls)

Generalized least squares fit by REML

Model: lat ~ group

Data: a

AIC BIC logLik

8398.323 8417.077 -4195.162

Variance function:

Structure: Different standard deviations per stratum

Formula: ~1 | group

Parameter estimates:

1 2

1.0000000 0.9007723

Coefficients:

Value Std.Error t-value p-value

(Intercept) -33.21418 5.126799 -6.478543 0.000

group 5.14453 3.151173 1.632576 0.103 # not significant

Correlation:

(Intr)

group -0.952

Standardized residuals:

Min Q1 Med Q3 Max

-1.2754865 -0.7927735 -0.6368558 1.1454067 1.6568280

Residual standard error: 46.98738

Degrees of freedom: 805 total; 803 residual

**Latitudinal distribution_R comparison_TOTAL**

# comparison between guadalupian and lopingian total data

> a<-read.table("pmed_psup.txt", header=TRUE, sep="\t")

> group <- as.factor(a[,2])

> vf2 <- varIdent(form=~1|group)

> M.lm <- gls(lat~group,data=a)

> M.gls <- gls(lat~group,data=a,weights=vf2)

> anova(M.lm,M.gls)

Model df AIC BIC logLik Test L.Ratio p-value

M.lm 1 3 4348.388 4360.614 -2171.194

M.gls 2 4 4349.537 4365.838 -2170.768 1 vs 2 0.8518153 0.356

> summary(M.lm)

Generalized least squares fit by REML

Model: lat ~ group

Data: a

AIC BIC logLik

4348.388 4360.614 -2171.194

Coefficients:

Value Std.Error t-value p-value

(Intercept) -33.96523 7.773988 -4.369088 0.0000

group -3.06017 4.214335 -0.726133 0.4681 # not significant

Correlation:

(Intr)

group -0.976

Standardized residuals:

Min Q1 Med Q3 Max

-0.7989006 -0.6462570 -0.5942323 1.1480061 2.1912457

Residual standard error: 35.17888

Degrees of freedom: 437 total; 435 residual

# comparison between lopingian and early triassic total data

> a<-read.table("psup_tinf.txt", header=TRUE, sep="\t")

> group <- as.factor(a[,2])

> M.lm <- gls(lat~group,data=a)

> vf2 <- varIdent(form=~1|group)

> M.gls <- gls(lat~group,data=a,weights=vf2)

> anova(M.lm,M.gls)

Model df AIC BIC logLik Test L.Ratio p-value

M.lm 1 3 8913.267 8927.555 -4453.634

M.gls 2 4 8886.037 8905.088 -4439.018 1 vs 2 29.23035 <.0001

> summary(M.gls)

Generalized least squares fit by REML

Model: lat ~ group

Data: a

AIC BIC logLik

8886.037 8905.088 -4439.018

Variance function:

Structure: Different standard deviations per stratum

Formula: ~1 | group

Parameter estimates:

1 2

1.000000 1.311375

Coefficients:

Value Std.Error t-value p-value

(Intercept) -60.37517 4.204186 -14.360726 0

group 20.28959 2.721634 7.454931 0 # significant

Correlation:

(Intr)

group -0.946

Standardized residuals:

Min Q1 Med Q3 Max

-1.3665293 -0.7850980 -0.5880915 0.9485102 2.2271995

Residual standard error: 34.61099

Degrees of freedom: 867 total; 865 residual

# comparison between early triassic and middle triassic total data

> a<-read.table("tinf_tmed.txt", header=TRUE, sep="\t")

> group <- as.factor(a[,2])

> vf2 <- varIdent(form=~1|group)

> M.lm <- gls(lat~group,data=a)

> M.gls <- gls(lat~group,data=a,weights=vf2)

> anova(M.lm,M.gls)

Model df AIC BIC logLik Test L.Ratio p-value

M.lm 1 3 11879.28 11894.43 -5936.640

M.gls 2 4 11862.92 11883.12 -5927.458 1 vs 2 18.36473 <.0001

> summary(M.gls)

Generalized least squares fit by REML

Model: lat ~ group

Data: a

AIC BIC logLik

11862.92 11883.12 -5927.458

Variance function:

Structure: Different standard deviations per stratum

Formula: ~1 | group

Parameter estimates:

1 2

1.0000000 0.8362516

Coefficients:

Value Std.Error t-value p-value

(Intercept) -28.762110 4.265760 -6.742553 0e+00

group 8.966134 2.498544 3.588544 3e-04 # significant

Correlation:

(Intr)

group -0.96

Standardized residuals:

Min Q1 Med Q3 Max

-1.7409773 -1.0239277 0.4170601 0.8320167 1.5288802

Residual standard error: 45.38799

Degrees of freedom: 1155 total; 1153 residual

**Latitudinal distribution_Trias detail**

# comparison between lopingian and induan skeletal data

> a<-read.table("lopind.txt", header=TRUE, sep="\t")

> group <- as.factor(a[,2])

> vf2 <- varIdent(form=~1|group)

> M.lm <- gls(lat~group,data=a)

> M.gls <- gls(lat~group,data=a,weights=vf2)

> anova(M.lm,M.gls)

Model df AIC BIC logLik Test L.Ratio p-value

M.lm 1 3 543.6990 550.6912 -268.8495

M.gls 2 4 545.0102 554.3331 -268.5051 1 vs 2 0.6888715 0.4065

> summary(M.lm)

Generalized least squares fit by REML

Model: lat ~ group

Data: a

AIC BIC logLik

543.699 550.6912 -268.8495

Coefficients:

Value Std.Error t-value p-value

(Intercept) 12.22168 2.750170 4.443974 0

group 10.85195 1.810383 5.994284 0 # significant

Correlation:

(Intr)

group -0.945

Standardized residuals:

Min Q1 Med Q3 Max

-3.0870529 -0.6364582 -0.2630507 0.7395964 1.4777595

Residual standard error: 7.928464

Degrees of freedom: 78 total; 76 residual

# comparison between induan and olenekian skeletal data

> a<-read.table("indole.txt", header=TRUE, sep="\t")

> group <- as.factor(a[,2])

> M.lm <- gls(lat~group,data=a)

> vf2 <- varIdent(form=~1|group)

> M.gls <- gls(lat~group,data=a,weights=vf2)

> anova(M.lm,M.gls)

Model df AIC BIC logLik Test L.Ratio p-value

M.lm 1 3 1574.723 1584.603 -784.3614

M.gls 2 4 1562.820 1575.993 -777.4101 1 vs 2 13.90263 2e-04

> summary(M.gls)

Generalized least squares fit by REML

Model: lat ~ group

Data: a

AIC BIC logLik

1562.82 1575.993 -777.4101

Variance function:

Structure: Different standard deviations per stratum

Formula: ~1 | group

Parameter estimates:

1 2

1.000000 1.772395

Coefficients:

Value Std.Error t-value p-value

(Intercept) 42.96788 2.69833 15.923881 0

group -9.04229 1.60406 -5.637131 0 # significant

Correlation:

(Intr)

group -0.957

Standardized residuals:

Min Q1 Med Q3 Max

-3.35071250 -0.66992727 0.03373132 0.83934703 1.58239815

Residual standard error: 7.304592

Degrees of freedom: 201 total; 199 residual

# comparison between olenekian and anisian skeletal data

>> a<-read.table("oleani.txt", header=TRUE, sep="\t")

> group <- as.factor(a[,2])

> vf2 <- varIdent(form=~1|group)

> M.lm <- gls(lat~group,data=a)

> M.gls <- gls(lat~group,data=a,weights=vf2)

> anova(M.lm,M.gls)

Model df AIC BIC logLik Test L.Ratio p-value

M.lm 1 3 2751.867 2763.353 -1372.933

M.gls 2 4 2752.752 2768.068 -1372.376 1 vs 2 1.114891 0.291

> summary(M.lm)

Generalized least squares fit by REML

Model: lat ~ group

Data: a

AIC BIC logLik

2751.867 2763.353 -1372.933

Coefficients:

Value Std.Error t-value p-value

(Intercept) 24.980987 2.328230 10.729604 0.0000

group -0.097693 1.462276 -0.066809 0.9468 # not significant

Correlation:

(Intr)

group -0.949

Standardized residuals:

Min Q1 Med Q3 Max

-1.70841058 -0.84155149 0.06411778 0.98276193 1.51557882

Residual standard error: 13.51741

Degrees of freedom: 342 total; 340 residual

# comparison between anisian and ladinian skeletal data

> a<-read.table("anilad.txt", header=TRUE, sep="\t")

> group <- as.factor(a[,2])

> M.lm <- gls(lat~group,data=a)

> vf2 <- varIdent(form=~1|group)

> M.gls <- gls(lat~group,data=a,weights=vf2)

> anova(M.lm,M.gls)

Model df AIC BIC logLik Test L.Ratio p-value

M.lm 1 3 1610.287 1620.152 -802.1435

M.gls 2 4 1608.183 1621.336 -800.0917 1 vs 2 4.103682 0.0428

> summary(M.gls)

Generalized least squares fit by REML

Model: lat ~ group

Data: a

AIC BIC logLik

1608.183 1621.336 -800.0917

Variance function:

Structure: Different standard deviations per stratum

Formula: ~1 | group

Parameter estimates:

1 2

1.0000000 0.7119719

Coefficients:

Value Std.Error t-value p-value

(Intercept) 18.9364 2.915979 6.494011 0.0000

group 5.8492 2.263523 2.584113 0.0105 # significant

Correlation:

(Intr)

group -0.947

Standardized residuals:

Min Q1 Med Q3 Max

-1.7031038 -0.8102075 0.4741937 0.9461585 1.3530156

Residual standard error: 14.04035

Degrees of freedom: 200 total; 198 residual

>

# comparison between lopingian and olenekian skeletal data

> a<-read.table("lopole.txt", header=TRUE, sep="\t")

> group <- as.factor(a[,2])

> M.lm <- gls(lat~group,data=a)

> vf2 <- varIdent(form=~1|group)

> M.gls <- gls(lat~group,data=a,weights=vf2)

> anova(M.lm,M.gls)

Model df AIC BIC logLik Test L.Ratio p-value

M.lm 1 3 1651.818 1661.845 -822.9090

M.gls 2 4 1642.995 1656.365 -817.4977 1 vs 2 10.82271 0.001

> summary(M.gls)

Generalized least squares fit by REML

Model: lat ~ group

Data: a

AIC BIC logLik

1642.995 1656.365 -817.4977

Variance function:

Structure: Different standard deviations per stratum

Formula: ~1 | group

Parameter estimates:

1 2

1.000000 1.545719

Coefficients:

Value Std.Error t-value p-value

(Intercept) 21.263979 2.716857 7.826684 0.0000

group 1.809657 1.611859 1.122714 0.2628 # not significant

Correlation:

(Intr)

group -0.957

Standardized residuals:

Min Q1 Med Q3 Max

-2.52079252 -0.83319112 0.06694463 0.71305924 1.58239814

Residual standard error: 8.375793

Degrees of freedom: 211 total; 209 residual

S2.2 Occupancy Ratio


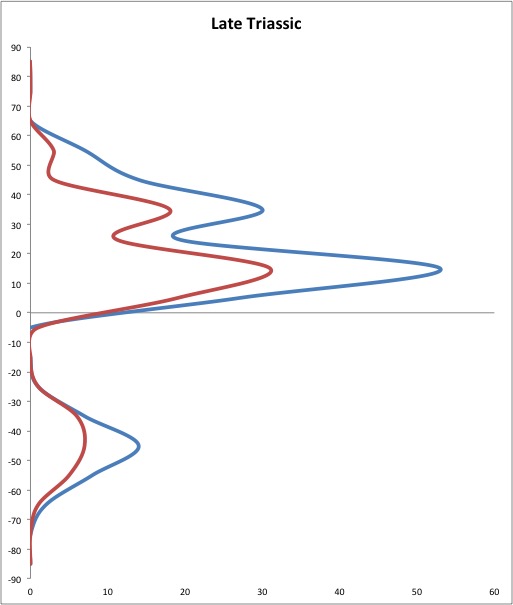


**Fig. S2 -1** Palaeolatitudinal distribution of AF (All Formations; in blue) and TBF (Tetrapod-bearing formations; in red) for the Late Triassic.

S3.3 Non Marine Area


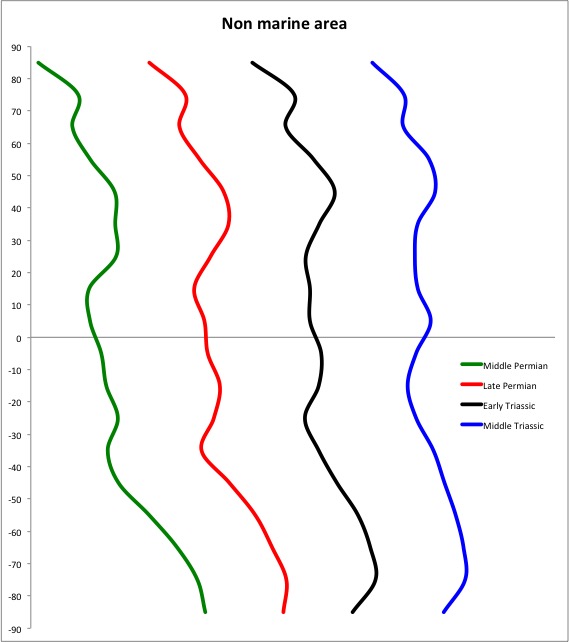


**Fig. S2 – 2** Palaeolatitudinal distribution of Non Marine Area
